# Head-to-head comparison of the safety of tocilizumab and tumor necrosis factor inhibitors in rheumatoid arthritis patients (RA) in clinical practice: results from the registry of Japanese RA patients on biologics for long-term safety (REAL) registry

**DOI:** 10.1186/s13075-015-0583-8

**Published:** 2015-03-23

**Authors:** Ryoko Sakai, Soo-Kyung Cho, Toshihiro Nanki, Kaori Watanabe, Hayato Yamazaki, Michi Tanaka, Ryuji Koike, Yoshiya Tanaka, Kazuyoshi Saito, Shintaro Hirata, Koichi Amano, Hayato Nagasawa, Takayuki Sumida, Taichi Hayashi, Takahiko Sugihara, Hiroaki Dobashi, Shinsuke Yasuda, Tetsuji Sawada, Kazuhiko Ezawa, Atsuhisa Ueda, Takao Fujii, Kiyoshi Migita, Nobuyuki Miyasaka, Masayoshi Harigai, for the REAL Study Group

**Affiliations:** Department of Pharmacovigilance, Graduate School of Medical and Dental Sciences, Tokyo Medical and Dental University, 1-5-45, Yushima, Bunkyo-ku, Tokyo 113-8519 Japan; Department of Rheumatology, Graduate School of Medical and Dental Sciences, Tokyo Medical and Dental University, 1-5-45, Yushima, Bunkyo-ku, Tokyo 113-8519 Japan; Department of Rheumatology, Hanyang University Hospital for Rheumatic Diseases, 222 Wangsimni-ro, Seongdong-gu, Seoul 133-791 South Korea; Clinical Research Center, Tokyo Medical and Dental University Hospital, 1-5-45, Yushima, Bunkyo-ku, Tokyo 113-8519 Japan; Global Center of Excellence (GCOE) Program, International Research Center for Molecular Science in Tooth and Bone Diseases, 1-5-45, Yushima, Bunkyo-ku, Tokyo 113-8519 Japan; The First Department of Internal medicine, University of Occupational and Environmental Health, 1-1, Iseigaoka, Kitakyushu Yahatanishi-ku, Fukuoka 807-0804 Japan; Department of Rheumatology/Clinical Immunology, Saitama Medical Center, Saitama Medical University, 1981 Kamoda, Kawagoe-shi, Saitama 350-8550 Japan; Division of Clinical Immunology, Doctoral Program in Clinical Sciences, Graduate School of Comprehensive Human Sciences, University of Tsukuba, 1-1-1, Tennodai, Tsukuba, Ibaraki 305-0006 Japan; Department of Rheumatology, Tokyo Metropolitan Geriatric Hospital, 35-2, Sakaecho, Itabashi-ku, Tokyo 173-0015 Japan; Department of Internal Medicine, Division of Hematology, Rheumatology and Respiratory Medicine, Faculty of Medicine, Kagawa University, 1750-1 Ikenobe, Miki-cho, Kita-gun, Kagawa 761-0793 Japan; Division of Rheumatology, Endocrinology and Nephrology, Hokkaido University Graduate School of Medicine, Kita 15. Nishi 7, Kita-ku, Sapporo, 060-8638 Japan; Department of Rheumatology, Tokyo Medical University, Nishi-Shinjuku 6-7-1, Shinjuku-ku, Tokyo 160-0023 Japan; Department of Internal Medicine, Kurashiki Kousai Hospital, 3542-1, Nakasho, Kurashiki-shi, Okayama 710-0016 Japan; Department of Internal Medicine and Clinical Immunology, Yokohama City University Graduate School of Medicine, 3-9, Fukuura, Kanazawa-ku, Yokohama, 236-0004 Japan; Department of the Control for Rheumatic Diseases, Graduate School of Medicine, Kyoto University, Yoshidakonoecho, Sakyo-ku, Kyoto 606-8501 Japan; Department of Rheumatology, National Hospital Organization Nagasaki Medical Center, 2-1001-1, Kubara, Omura, Nagasaki 856-8562 Japan

## Abstract

**Introduction:**

The objective of this study was to directly compare the safety of tocilizumab (TCZ) and TNF inhibitors (TNFIs) in rheumatoid arthritis (RA) patients in clinical practice.

**Methods:**

This prospective cohort study included RA patients starting TCZ [TCZ group, n = 302, 224.68 patient-years (PY)] or TNFIs [TNFI group, n = 304, 231.01 PY] from 2008 to 2011 in the registry of Japanese RA patients on biologics for long-term safety registry. We assessed types and incidence rates (IRs) of serious adverse events (SAEs) and serious infections (SIs) during the first year of treatment. Risks of the biologics for SAEs or SIs were calculated using the Cox regression hazard analysis.

**Results:**

Patients in the TCZ group had longer disease duration (*P* <0.001), higher disease activity (*P* = 0.019) and more frequently used concomitant corticosteroids (*P* <0.001) than those in the TNFI group. The crude IR (/100 PY) of SIs [TCZ 10.68 vs. TNFI 3.03; IR ratio (95% confidence interval [CI]), 3.53 (1.52 to 8.18)], but not SAEs [21.36 vs. 14.72; 1.45 (0.94 to 2.25)], was significantly higher in the TCZ group compared with the TNFI group. However, after adjusting for covariates using the Cox regression hazard analysis, treatment with TCZ was not associated with higher risk for SAEs [hazard ratio (HR) 1.28, 95% CI 0.75 to 2.19] or SIs (HR 2.23, 95% CI 0.93 to 5.37).

**Conclusions:**

The adjusted risks for SAEs and SIs were not significantly different between TCZ and TNFIs, indicating an influence of clinical characteristics of the patients on the safety profile of the biologics in clinical practice.

**Electronic supplementary material:**

The online version of this article (doi:10.1186/s13075-015-0583-8) contains supplementary material, which is available to authorized users.

## Introduction

Tocilizumab (TCZ), which is a humanized antibody against the interleukin 6 (IL-6) receptor [1], inhibits signaling mediated by IL-6 [2] and was first approved to treat rheumatoid arthritis (RA) in Japan in 2008. The superior efficacy of TCZ compared to a control drug or placebo in RA patients has been demonstrated by a series of clinical trials [3-12]. In clinical practice, TCZ showed excellent effectiveness in patients with established RA [13]. Safety profiles of TCZ in patients with RA were clarified by the Japanese post-marketing surveillance (PMS) program [14] and a meta-analysis [15]. In the PMS of TCZ, the most frequent category of serious adverse events (SAEs) was infection and the most common infection was pneumonia. The incidence rate (IR) of serious infections (SIs) per 100 patient-years (PY) was 9.1, with older age, longer disease duration, respiratory diseases, and prednisolone dose ≥5 mg/day at baseline identified as significant risk factors for development of SIs during the first six months of treatment with TCZ [14]. The favorable benefit-risk balance of TCZ has led to the worldwide use of this biologic for treating RA [16].

In 2013, the European League Against Rheumatism recommendations for the management of RA were updated [17]. They now express no preference for the use of a specific biological agent; this indicates that TCZ, as well as tumor necrosis factor inhibitors (TNFIs) and abatacept, can be first line biologics. Therefore, for the clinical selection of biologics, it is necessary to compare the efficacy and safety of TCZ with those of other biologics. Systematic reviews [18,19] and meta-analyses [20] indirectly comparing efficacy of TCZ with other biologics showed that TCZ had similar response rates in patients with RA. Results from a clinical trial or study comparing TCZ with another biologic have been reported. Gabay *et al.* demonstrated that TCZ monotherapy was superior to adalimumab monotherapy in RA patients who are intolerant to methotrexate [21]. A Danish registry reported the comparison of effectiveness between TCZ and abatacept (ABA) [22] and found that declines in disease activity during 48 weeks were similar between the drugs.

There are few data comparing the safety of TCZ with other biologics. A meta-analysis found no significant difference in the risk of SIs between TCZ and other biologics [23]. Using a Japanese single institution registry with a relatively small number of patients, Yoshida *et al.* reported the safety profiles of TCZ and TNFIs; IRs of SAE were 15.9/100 PY in the TCZ group and 13.9/100PY in the TNFI group [24]. However, to date, no detailed comparison of SAEs between TCZ and TNFIs, particularly the types and incidence of SIs, has been reported. Additional direct observational studies are needed to clarify the risk of use of TCZ versus TNFIs for the development of SAEs and SIs in clinical practice.

In this study, we utilized the database of the registry of Japanese RA patients on biologics for long-term safety (REAL), a prospective, multi-center cohort with a large number of patients, and herein report IRs for each category of SAEs for TCZ with hazard ratios (HRs) for SAEs and SIs from the use of TCZ compared to the use of TNFIs.

## Methods

### Database

The REAL is a prospective cohort established to investigate the long-term safety of biologics in RA patients. Details of the REAL have been previously described [25]. In brief, 27 institutions participate in the REAL, including 16 university hospitals and 11 referring hospitals. The criteria for enrollment in the REAL include patients meeting the 1987 American College of Rheumatology criteria for RA [26], written informed consent, and starting or switching treatment with biologics or starting, adding or switching non-biologics at the time of enrollment in the study. Enrollment in the REAL database was started in June 2005 and closed in January 2012. Data were retrieved from the REAL database on 5 March 2012 for this study. This study was in compliance with the Helsinki Declaration (revised in 2008). The REAL study was approved by the ethics committees of the Tokyo Medical and Dental University Hospital and all other participating institutions. All ethical bodies that approved this study are shown in the Acknowledgements section.

### Data collection

Recorded baseline data for each patient includes demography, disease activity, physical disability, comorbidities, treatments, and laboratory data at the beginning of the observation period. A follow-up form was submitted every six months to the REAL Data Center at the Department of Pharmacovigilance of Tokyo Medical and Dental University by site investigators to report the occurrence of SAEs, current RA disease activity, treatments, and clinical laboratory data [25]. Steinbrocker’s classification [27] was used as the baseline measurement for the physical disability of each patient instead of the Health Assessment Questionnaire Disability Index [28]. The investigators in each hospital confirmed the accuracy of their data submitted to the REAL Data Center. The center examined all data sent by site investigators and made inquiries if needed to verify accuracy of the data.

### Patients

**Figure 1:**
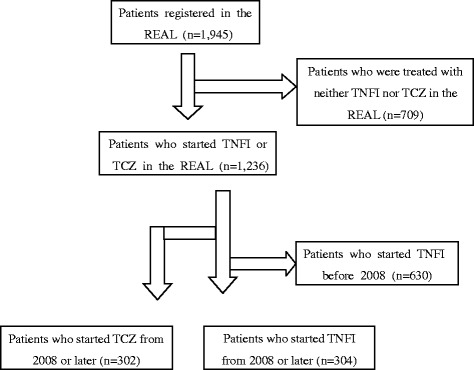
**Flow chart of rheumatoid arthritis (RA) patients enrolled in this study from the REAL.**

A flow chart of patients enrolled in this study from the REAL is shown in Figure 1. By March 2012, 1,945 patients with RA were registered in the REAL. Of 1,236 patients who started infliximab (IFX), etanercept (ETN), adalimumab (ADA) or TCZ at the time of enrollment or after enrollment in the REAL, we identified 302 patients who started TCZ (TCZ group). Patients who used both TCZ and TNFIs at different periods were assigned to the TCZ group. We then excluded 630 patients who had started any of the TNFIs before 2008 because TCZ was approved for RA in Japan in 2008, and identified 304 patients who started only TNFIs between 2008 and 2011 (TNFI group). The first TNFI of each patient in the TNFI group was IFX for 117 patients, ETN for 80, and ADA for 107. No patients started abatacept, golimumab, or certolizumab pegol in the REAL during the time our data were compiled for this study.

### Follow-up

For patients in the TCZ group, the start date for the observation period was the date TCZ was first administered. For patients in the TNFI group, the start of the observation period was the date of the first administration of TNFI from 2008 to 2011. Observation was ended at either 1.0 year after the start of the observation period, or on the date of death of a patient, loss to follow up, enrollment in clinical trials, when therapy with a biologic of interest was discontinued for more than 90 days, or on 5 March 2012, whichever came first. The period following switching to another biologic was excluded from this study. The date of the last administration of each biologic was retrieved from medical records and reported by the site investigators.

### Definition of serious adverse events

Our definition of a SAE, including SIs, was based on the report by the International Conference on Harmonization [29]. In addition, bacterial infections that required intravenous administration of antibiotics and opportunistic infections were regarded as SAEs [30,31]. SAEs were classified using the System Organ Class (SOC) of the medical dictionary for regulatory activities (MedDRA version 16.0).

### Statistical analysis

The chi-square test for categorical variables and Student’s t-test or the Mann–Whitney test for continuous variables were used for comparisons among groups. The IR per 100 PY and incidence rate ratios (IRR) with their 95% confidence intervals (CIs) were calculated. Kaplan-Meier methods and log-rank tests were used to compare drug retention rates between the groups. The Cox regression hazard model with the forced entry method was used to calculate HRs of use of TCZ versus TNFIs for SAEs and SIs. As a sensitivity analysis, we performed the same analysis in patients treated with methotrexate (MTX) at baseline, considering substantial differences in clinical characteristics between MTX users and non-users. These statistical analyses were conducted using SPSS (version 20.0, SPSS Inc., Chicago, IL, USA). All *P* values were two-tailed and *P* <0.05 was considered statistically significant.

## Results

### Demographic and clinical baseline characteristics of patients

Baseline data for the patients are shown in Table 1. Compared to the TNFI group, the TCZ group had longer disease duration (*P* <0.001), higher disease activity (*P* = 0.019), more advanced disease stage (*P* <0.001), and poorer physical function (*P* = 0.005). Age did not differ significantly between the groups. A significantly higher rate of the patients in the TCZ group had received three or more non-biological disease-modifying anti-rheumatic drugs before starting the biologic (*P* = 0.034), was biologic non-naive (*P* <0.001), and was treated with oral corticosteroids (*P* <0.001). The proportion of patients treated with MTX in the TCZ group was significantly (*P* < 0.001) lower than in the TNFI group (n = 160 (53.0%) versus n = 260 (85.5%)). We also compared characteristics of MTX users at baseline. Patients in the TCZ group had significantly longer disease duration (*P* = 0.003), more advanced stage (*P* = 0.005) and poorer physical function (*P* = 0.042) than those in the TNFI group (Additional file 1: Table S1).

**Table 1:** **Demographic and clinical characteristics of RA patients treated with TCZ or TNFIs**

**Characteristics**	**TCZ group (number = 302)**	**TNFI group (number = 304)**	***P*** **value**
Age, years	59.20 ± 13.04	57.33 ± 15.18	0.275
Female, %	82.5	82.8	0.425
Disease duration, years	10.20 ± 8.64	7.96 ± 8.70	<0.001
Steinbrocker’s stage^a^	51.0	35.2	<0.001
(III or IV), %			
Steinbrocker’s class^a^	29.1	19.4	0.005
(3 or 4), %			
Previous biologic use, %	70.5	10.5	<0.001
Number of previous non-biological DMARDs ≥3, %	47.0	38.5	0.034
DAS28CRP (3)^b^	4.50 ± 1.23 (n = 233)	4.25 ± 1.24 (n = 279)	0.019
Pulmonary diseases^c^, %	20.2	15.5	0.128
Diabetes mellitus, %	10.9	10.5	0.873
Liver diseases^d^, %	6.6	4.6	0.281
Kidney diseases^e^, %	3.6	0.7	0.011
MTX use, %	53.0	85.5	<0.001
MTX dose, mg/week	8.41 ± 2.80	8.54 ± 2.28	0.237
Oral corticosteroids use, %	65.6	51.0	<0.001
PSL-equivalent dose^f^, mg/day	5.32 ± 3.19	4.99 ± 3.05	0.433

^a^Steinbrocker’s classification was used to define RA disease stages and classes; ^b^DAS28CRP (3) was calculated based on three variables: swollen and tender 28-joint counts and CRP; ^c^pulmonary diseases included interstitial lung disease, chronic obstructive pulmonary disease, and asthma; ^d^liver diseases included hepatitis B carrier, hepatitis C carrier, fatty liver, hepatitis, primary biliary cirrhosis, positive anti-hepatitis C antibody, cholelithiasis, and abnormal liver function tests; ^e^kidney diseases included nephrotic syndrome, nephritis, renal failure, chronic kidney disease, renal hypertension, hemi-kidney, and elevation of serum creatinine; ^f^the oral corticosteroids dose was converted to the equivalent prednisolone dosage. CRP; C-reactive protein; DAS28, disease activity score including 28-joint count; DMARDs, disease-modifying antirheumatic drugs; MTX, methotrexate; PSL, prednisolone; RA, rheumatoid arthritis; TCZ, tocilizumab; TNFIs, tumor necrosis factor inhibitors.

### Retention rates for TCZ and TNFIs

**Figure 2:**
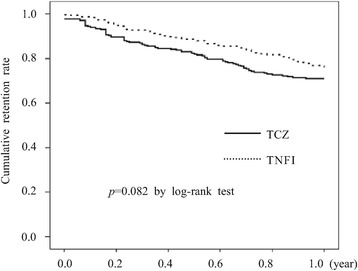
**Kaplan-Meier curves for time to discontinuation for each group.** Drug retention rates were compared using the log-rank test between tocilizumab (TCZ) and tumor necrosis factor inhibitors (TNFIs). The y axis shows the cumulative retention rates.

The median (interquartile (IQR)) treatment period was 1.00 (0.50 to 1.00) year for the TCZ group and 1.00 (0.51 to 1.00) year for the TNFI group. The number of patients who discontinued biologics for any reason during the observation period was 81 (26.8%) in the TCZ group and 62 (20.4%) in the TNFI group, not a significant difference (*P* = 0.062 by chi-square). The development of AEs was the most frequent reason for discontinuation in both the TCZ group (n = 41, 50.6%) and the TNFI group (n = 24, 38.7%). There was no significant difference in the retention rates of the biologics for one year between the two groups (71.0% in the TCZ group, 76.1% in the TNFI group, *P* = 0.082 by Kaplan-Meier analysis and log-rank test) (Figure 2).

### Types and occurrence of SAEs

The IRs for SAEs are summarized in Table 2. Among the 606 patients, 82 SAEs were reported during the observation period; 48 in the TCZ group and 34 in the TNFI group. The crude IRR, comparing the TCZ group with the TNFI group for SAEs, was 1.45 (95% CI, 0.94 to 2.25) and for SIs was 3.53 (95% CI, 1.52 to 8.18). There were no significant differences in the IR of SAEs among the three TNFIs (data not shown). For patients using MTX at baseline, the IR of SAEs in the TCZ group was not significantly higher than that in the TNFI group (IRR 1.48 95% CI, 0.85 to 2.61), whereas, the IR of SIs was significantly higher in the TCZ group compared to the TNFI group (IRR 2.88 95% CI, 1.13 to 7.32). The IR of SIs in the TCZ group was significantly higher than that in the TNFI group in patients with previous biologics exposure (4.4 (1.7 to 11.6)), but not for SAEs (1.6 (0.8 to 3.0)).

**Table 2:** **Occurrence of SAEs in patients with RA treated with TCZ or TNFIs** ^**a**^

**Types of SAEs**	**TCZ group, 224.68 PY**	**TNFI group, 231.01 PY**	**TCZ versus TNFI**
	**IR (/100PY)**	**IR (/100PY)**	**Crude IRR (95% CI)**
Total SAEs	21.36 (15.94 to 28.07)	14.72 (10.37 to 20.32)	1.45 (0.94 to 2.25)
Serious infection (SI)	10.68 (7.02 to 15.63)	3.03 (1.35 to 5.95)	3.53 (1.52 to 8.18)
Pulmonary infection	3.12 (1.39 to 6.12)	1.30 (0.36 to 3.46)	2.40 (0.62 to 9.28)
Non-pulmonary infection	7.57 (4.57 to 11.84)	1.73 (0.58 to 4.12)	4.37 (1.47 to 12.99)
Skin infection	1.78 (0.60 to 4.23)	0.43 (0.04 to 2.02)	4.11 (0.46 to 36.80)
Urinary tract infection	0.89 (0.18 to 2.85)	0.43 (0.04 to 2.02)	2.06 (0.19 to 22.68)
Gastrointestinal infection	0.89 (0.18 to 2.85)	0.43 (0.04 to 2.02)	2.06 (0.19 to 22.68)
Bone and joint infections	2.23 (0.84 to 4.88)	0	NA
Sepsis	1.34 (0.37 to 3.56)	0	NA
Other infection	0.45 (0.04 to 2.08)	0.43 (0.04 to 2.02)	1.03 (0.06 to 16.44)
Pulmonary disease, except infection	2.23 (0.84 to 4.88)	2.16 (0.82 to 4.74)	1.03 (0.30 to 3.55)
Cardiovascular or cerebrovascular disease	0.45 (0.04 to 2.08)	2.16 (0.82 to 4.74)	0.21 (0.02 to 1.76)
Malignancy	0.89 (0.18 to 2.85)	1.30 (0.36 to 3.46)	0.69 (0.11 to 4.10)
Death	1.78 (0.60 to 4.23)	0.87 (0.17 to 2.78)	2.06 (0.38 to 11.23)
Others	7.12 (4.24 to 11.29)	6.06 (3.47 to 9.90)	1.18 (0.57 to 2.41)

^a^Crude incidence rate per 100 PYs and crude incidence rate ratio with their 95% CI were calculated for each category of serious adverse events. CI: confidence interval; IR: incidence rate; IRR: incidence rate ratio; NA: not applicable; PY: patient-year; RA: rheumatoid arthritis; SAEs: serious adverse events; TCZ: tocilizumab; TNFIs: tumor necrosis factor inhibitors.

In the TCZ group, 53% of patients had received MTX at baseline; there were no significant differences in the unadjusted IR of SAEs and SIs between MTX users and non-users (data not shown). In the TCZ group, 70% of the patients had previously used biologics; these patients had safety profiles similar to the biologics-naïve patients (data not shown).

In the TCZ group, there were 24 SIs including five cases of opportunistic infections (herpes zoster (two); Pneumocystis pneumonia (PCP) (one); pulmonary aspergillosis (one); and esophageal candidiasis (one)) and 19 non-opportunistic infections. In the TNFI group, of seven cases with SIs, three were opportunistic infections (herpes zoster (one); PCP (one); and pulmonary cryptococcosis (one)), and four non-opportunistic infections. The respiratory system was the most frequent site of infection in both groups (TCZ (seven) and TNFI (three)), followed in the TCZ group by five in bone and joints and four in skin and subcutaneous tissue. There were no significant differences in the IR for pulmonary infection (IRR 2.40 95% CI, 0.62 to 9.28), but the IR for non-pulmonary infections was significantly higher in the TCZ group compared to the TNFI group (IRR 4.37 95% CI, 1.47 to 13.0). One perforation of the upper gastrointestinal tract developed in the TCZ group. No anaphylactic reactions were reported in either group.

### Evaluation of risk of TCZ for development of SAEs compared to TNFI

We compared patients who had and had not experienced SAEs using a univariate analysis and selected variables for the multivariate Cox regression hazard analysis to evaluate the risk of the use of TCZ for the development of a SAE. After adjusting for age, gender, disease activity score including 28-joint count C-reactive protein (3), comorbidity, use of oral corticosteroids (prednisolone-equivalent dose) ≥5 mg/day, and Steinbrocker’s class, the hazard ratio (HR) of the use of TCZ compared to the use of TNFI for developing SAEs was 1.28 (95% CI, 0.75 to 2.19, *P* = 0.370), not significantly elevated (Table 3). Significant risk factors influencing the development of SAEs were age by decade (HR 1.47, 95% CI, 1.15 to 1.88, *P* = 0.002), the presence of a comorbidity (HR 1.86, 95% CI, 1.07 to 3.24, *P* = 0.029), and the use of oral corticosteroids (prednisolone-equivalent dose) ≥5 mg/day (HR 1.72, 95% CI, 1.01 to 2.93, *P* = 0.047) (Table 3). We evaluated the risk of use of TCZ for development of SAEs in patients given MTX at baseline as a sensitivity analysis, and found the HR of use of TCZ was 1.21 (0.55 to 2.65, *P* = 0.632) compared to the use of TNFI (Table 3).

**Table 3:** **Factors influencing development of SAEs in patients with RA treated with TCZ or TNFIs** ^**a**^

**Variable**	**All patients**		**MTX users**	
	**HR (95% CI)** ^**c**^	***P*** **value** ^**c**^	**HR (95% CI)** ^**d**^	***P*** **value** ^**d**^
Age by decade	1.47 (1.15 to 1.88)	0.002	1.58 (1.07 to 2.35)	0.022
Female	0.74 (0.40 to 1.38)	0.345	0.96 (0.38 to 2.47)	0.940
DAS28CRP (3)	1.06 (0.98 to 1.14)	0.151	1.06 (0.76 to 1.48)	0.744
Comorbidity^b^	1.86 (1.07 to 3.24)	0.029	2.10 (0.92 to 4.79)	0.077
PSL ≥5 (mg/day)	1.72 (1.01 to 2.93)	0.047	1.64 (0.74 to 3.63)	0.223
Steinbrocker’s Class 3 or 4	1.37 (0.77 to 2.43)	0.287	1.10 (0.47 to 2.60)	0.825
Tocilizumab	1.28 (0.75 to 2.19)	0.370	1.21 (0.55 to 2.65)	0.632

^a^Cox regression analysis with the independent variables included in the Table; ^b^comorbidity included pulmonary diseases, diabetes mellitus, liver diseases, and kidney diseases; ^c^Cox regression analysis was applied in all patients; ^d^Cox regression analysis was applied in patients who were treated with MTX at baseline. CI: confidence interval; CRP: C-reactive protein; DAS28CRP (3): 3-variable disease activity score including 28-joint count; HR: hazard ratio; MTX: methotrexate; PSL: prednisolone RA: rheumatoid arthritis; SAEs: serious adverse events; TCZ: tocilizumab; TNFIs: tumor necrosis factor inhibitors.

### Evaluation of risk of TCZ for development of SIs compared to TNFIs

We next investigated the risk of use of TCZ compared to the use of TNFI for development of SIs. After comparing patients who had and had not experienced SIs using a univariate analysis, we selected adjusting factors for the multivariate analysis. The HRs for using TCZ compared with TNFI were 2.23 (95% CI, 0.93 to 5.37; *P* = 0.074) in all the patients and 1.93 (95% CI, 0.72 to 5.17; *P* = 0.190) in patients treated with MTX at baseline (Table 4). The use of oral corticosteroids (prednisolone-equivalent dose) ≥5 mg/day was a significant risk factor influencing the development of SIs (HR 2.26, 95% CI, 1.02 to 5.01, *P* = 0.046).

**Table 4:** **Factors influencing development of SI in patients with RA treated with TCZ or TNFIs** ^**a**^

**Variable**	**All patients**		**MTX users**	
	**HR (95% CI)** ^**c**^	***P*** **value** ^**c**^	**HR (95% CI)** ^**d**^	***P*** **value** ^**d**^
Age by decade	1.34 (0.95 to 1.89)	0.093	1.31 (0.86 to 2.00)	0.210
Female	3.27 (0.77 to 13.98)	0.110	2.20 (0.49 to 9.93)	0.305
Comorbidity^b^	2.20 (0.95 to 5.11)	0.067	2.49 (0.87 to 7.10)	0.088
PSL ≥5 (mg/day)	2.26 (1.02 to 5.01)	0.046	2.04 (0.77 to 5.44)	0.154
Tocilizumab	2.23 (0.93 to 5.37)	0.074	1.93 (0.72 to 5.17)	0.190

^a^Cox regression hazard models were performed using the independent variables included in the Table; ^b^comorbidity included pulmonary diseases, diabetes mellitus, liver diseases, and kidney diseases; ^c^Cox regression analysis was applied in all patients; ^d^Cox regression analysis was applied in patients who were treated with MTX at baseline. CI: confidence interval; HR: hazard ratio; MTX: methotrexate; PSL: prednisolone; RA: rheumatoid arthritis; SI: serious infection; TCZ: tocilizumab; TNFIs: tumor necrosis factor inhibitors.

## Discussion

In this study, we conducted a direct comparison of the safety of TCZ with TNFIs in clinical practice, using a prospective, multi-center cohort with the largest possible number of patients. We demonstrated that the unadjusted IR of SAEs was not significantly higher in the TCZ group compared with the TNFI group, whereas the unadjusted IR of SIs of the TCZ group was 3.5-fold higher than the TNFI group. However, after adjusting for covariates, the use of TCZ compared to the use of TNFIs was not significantly associated with the development of SAEs or SIs.

Some studies have investigated the safety of TCZ in RA patients [4,13,15,32-35]. It has been reported that the IR of SAEs was 20 to 30/100PY and that the most frequent SAE was infection (5 to 9/100PY) [4,14,15,36]. In the present study, the IRs of SAEs (21.36/100PY) and SIs (10.68/100PY) were similar to those of previous reports. The most frequently reported category of SAE in our study was infection and the incidence rate of non-pulmonary infection in the TCZ group was conspicuously higher compared to the TNFI group (7.57/100PY versus 1.73/100PY). Among non-pulmonary infections, skin and bone and joints were common sites in the TCZ group. Previous studies also reported that skin infections, as well as pulmonary infections, were frequently observed in patients treated with TCZ [4,15,24,33,37,38]. Although the reasons for the high incidence rates of these types of infections in patients given TCZ have not been explained, special attention should be paid, not only to pulmonary infections, but also to skin infections in TCZ users.

We found no increased risk for the use of TCZ compared to the use of TNFIs for the development of SIs after adjusting for covariates at baseline. It is notable that the unadjusted IR of SIs in the TCZ group (10.7 (7.02 to 15.6)) was significantly increased compared to the TNFI group (3.53 95%CI, 1.52 to 8.18); this can be explained by several factors. The multivariate analysis indicated that the differences in clinical characteristics of the patients between the two groups influenced the difference in IRs of SIs (Table 4). The use of oral corticosteroids (prednisolone-equivalent dose) ≥5 mg/day was a significant risk factor for SIs in our study. Previous studies have reported that use of oral corticosteroids significantly increased the risk of SIs in patients undergoing treatment with biologics [29,39,40]. Patients in the TCZ group of our study used concomitant corticosteroids more frequently. It has been shown that the presence of comorbidities increased the risk of SIs in RA patients [41]. Although the HR of comorbidities was 2.20 in our study, it did not achieve statistical significance (*P* = 0.067). Relatively more patients in the TCZ group than in the TNFI group had at least one comorbidity (34.1% for the TCZ group, 27.3% for the TNFI group, *P* = 0.069). These data indicate that patients in the TCZ group may be more predisposed to infections than those in the TNFI group.

The low IR of SIs in the TNFI group apparently contributed to the increased IRR of SIs. The IR of SIs in the TNFI group in our study (3.03/100 PY) was lower than in previous studies (5 to 6/100PY) [25,29,42,43], resulting in an increased IRR when comparing TCZ and TNFIs. We previously reported a significant decrease over time of the risk for SIs with TNFI treatment, possibly explained by evidence-based risk management of RA patients given TNFIs [44]. In the present study, patients in the TNFI group started TNFIs in or after 2008, five years after the approval of IFX for RA in Japan. Information about risk of SIs in patients given TNFIs from observational studies has been extensively shared among Japanese rheumatologists, leading to improved risk management and, in consequence, lowered IRs for SIs in the TNFI group [44]. To accurately compare the outcome between a new drug and an existing one, differences in the calendar year of drug approval should be considered. Therefore, in our study, we compared the use of two biologics, TCZ and TNFIs, in clinical practice during the same time period.

There are potential limitations of this study. First, we have to mention the possibility of selection bias. The patients in this study were enrolled from university hospitals or referral hospitals that are dedicated to the treatments of RA, which may indicate unidentified selection bias. However, because almost all patients who were registered from the participating hospitals to the all-cases post-marketing surveillance programs for each biological DMARD were enrolled in the REAL, selection bias was substantially low. Second, although there is concern about information bias, such as recall bias and reporting bias, in epidemiological studies in general, we collected patient data using the same case report form prospectively, which should overcome the misclassification and underestimation of SAEs derived from these types of bias. Third, clinical practice is always accompanied by the indication bias occurring when a drug is preferentially prescribed to patients with different baseline characteristics. In this study, it was notable that the difference in the percentage of patients who were given MTX at baseline between the two groups was significant, which would have affected the results of our study. To address this possibility, we estimated the risk of SAEs and SIs in patients with concomitant MTX in addition to the whole study population, and found them to be similar. Fourth, we did not investigate the comparison of effectiveness between the two groups due to incomplete data about disease activity in some patients.

## Conclusions

The adjusted risks for SAEs and SIs between TCZ and TNFI were not significantly different in clinical practice, although significantly higher IRs for SIs were observed in the TCZ group, possibly attributable to more infection susceptible clinical characteristics of the patients in the TCZ group.

## Additional file

Additional file 1:
**Demographic and clinical characteristics of RA patients treated with methotrexate at baseline.** This file provides demographic and clinical characteristics of RA patients given methotrexate at baseline in this study.

## Abbreviations

ABA: abatacept
ADA: adalimumab
CI: confidence interval
CRP: C-reactive protein
DAS28: disease activity score including 28-joint count
ETN: etanercept
HR: hazard ratio
IFX: infliximab
IL: interleukin
IQR: interquartile range
IR: incidence rate
IRR: incidence rate ratio
MTX: methotrexate
PCP: *Pneumocystis jirovecii* pneumonia
PMS: post-marketing surveillance
PSL: prednisolone
PY: patient-year
RA: rheumatoid arthritis
REAL: Japanese Rheumatoid Arthritis Patients on biologics for Long-Term Safety
SAE: serious adverse event
SI: serious infection
TCZ: tocilizumab
TNFI: tumor necrosis factor inhibitors
