# Supplementary material for: Head-to-head comparison of the safety of tocilizumab and tumor necrosis factor inhibitors in rheumatoid arthritis patients (RA) in clinical practice: results from the registry of Japanese RA patients on biologics for long-term safety (REAL) registry
Source: Arthritis Res Ther. 2015 Mar 23;17(1):74. doi: 10.1186/s13075-015-0583-8 (PMC4384380; doi:10.1186/s13075-015-0583-8)
Supplement: Additional file 1: — Demographic and clinical characteristics of RA patients treated with methotrexate at baseline. This file provides demographic and clinical characteristics of RA patients given methotrexate at baseline in this study. [file 13075_2015_583_MOESM1_ESM.pdf]

Supplementary Table 1. Demographic and clinical characteristics of RA patients treated with methotrexate at baseline

	**TCZ group (n=160)**	**TNFI group (n=260)**	***p* value**
Age, years	58.03 ± 12.35	56.50 ± 15.10	0.564
Female, %	85.6	82.3	0.373
Disease duration, years	9.22 ± 7.68	8.05 ± 8.84	0.003
Steinbrocker's Stage* (III or IV), %	49.4	35.4	0.005
Steinbrocker's Class* (3 or 4), %	26.9	18.5	0.042
Previous biologic use, %	77.5	10.8	<0.001
Number of previous non-biological DMARDs ≥3, %	42.5	36.5	0.223
DAS28CRP (3)**	4.39 ± 1.19 (n=118)	4.25 ± 1.24 (n=238)	0.235
Pulmonary diseases †, %	18.1	12.7	0.127
Diabetes mellitus, %	10.0	9.2	0.794
Liver diseases ‡, %	4.4	3.8	0.789
Kidney diseases §, %	1.9	0.8	0.284

MTX dose, mg/week	8.41 ± 2.80	8.54 ± 2.28	0.237
Oral corticosteroids use, %	68.8	50.0	<0.001
PSL-equivalent dose ¶, mg/day	5.25 ± 2.92	4.91 ± 3.20	0.261

RA: rheumatoid arthritis; TCZ: tocilizumab; TNF: tumor necrosis factor; DMARDs: disease-modifying antirheumatic drugs; DAS28: disease activity score including 28-joint count; CRP: C-reactive protein; MTX: methotrexate; PSL: prednisolone

*Steinbrocker's classification was used to define RA disease stages and classes.

**DAS 28CRP (3) was calculated based on three variables; swollen and tender 28-joint counts and CRP.

†Pulmonary diseases included interstitial lung disease, chronic obstructive pulmonary disease, and asthma.

‡Liver diseases included hepatitis B carrier, hepatitis C carrier, fatty liver, hepatitis, primary biliary cirrhosis, positive anti-hepatitis C antibody, cholelithiasis, and abnormal liver function tests.

§Kidney diseases included nephrotic syndrome, nephritis, renal failure, chronic kidney disease, renal hypertension, hemi-kidney, and elevation of serum creatinine.

¶The oral corticosteroids dose was converted to the equivalent prednisolone dosage.
